# Children of Low Socioeconomic Status Show Accelerated Linear Growth in Early Childhood; Results from the Generation R Study

**DOI:** 10.1371/journal.pone.0037356

**Published:** 2012-05-23

**Authors:** Lindsay M. Silva, Lenie van Rossem, Pauline W. Jansen, Anita C. S. Hokken-Koelega, Henriëtte A. Moll, Albert Hofman, Johan P. Mackenbach, Vincent W. V. Jaddoe, Hein Raat

**Affiliations:** 1 The Generation R Study Group, Erasmus MC, University Medical Center Rotterdam, Rotterdam, The Netherlands; 2 Department of Pediatrics, Erasmus MC, University Medical Center Rotterdam, Rotterdam, The Netherlands; 3 Julius Center for Health Sciences and Primary Care, University Medical Center Utrecht, Utrecht, The Netherlands; 4 Department of Child and Adolescent Psychiatry, Erasmus MC, University Medical Center Rotterdam, Rotterdam, The Netherlands; 5 Department of Epidemiology, Erasmus MC, University Medical Center Rotterdam, Rotterdam, The Netherlands; 6 Department of Public Health, Erasmus MC, University Medical Center Rotterdam, Rotterdam, The Netherlands; John Hopkins Bloomberg School of Public Health, United States of America

## Abstract

**Objectives:**

People of low socioeconomic status are shorter than those of high socioeconomic status. The first two years of life being critical for height development, we hypothesized that a low socioeconomic status is associated with a slower linear growth in early childhood. We studied maternal educational level (high, mid-high, mid-low, and low) as a measure of socioeconomic status and its association with repeatedly measured height in children aged 0–2 years, and also examined to what extent known determinants of postnatal growth contribute to this association.

**Methods:**

This study was based on data from 2972 mothers with a Dutch ethnicity, and their children participating in The Generation R Study, a population-based cohort study in Rotterdam, the Netherlands (participation rate 61%). All children were born between April 2002 and January 2006. Height was measured at 2 months (mid-90% range 1.0–3.9), 6 months (mid-90% range 5.6–11.4), 14 months (mid-90% range 13.7–17.9) and 25 months of age (mid-90% range 23.6–29.6).

**Results:**

At 2 months, children in the lowest educational subgroup were shorter than those in the highest (difference: −0.87 cm; 95% CI: −1.16, −0.58). Between 1 and 18 months, they grew faster than their counterparts. By 14 months, children in the lowest educational subgroup were taller than those in the highest (difference at 14 months: 0.40 cm; 95% CI: 0.08,0.72). Adjustment for other determinants of postnatal growth did not explain the taller height. On the contrary, the differences became even larger (difference at 14 months: 0.61 cm; 95% CI: 0.26,0.95; and at 25 months: 1.00 cm; 95% CI: 0.57,1.43)

**Conclusions:**

Compared with children of high socioeconomic status, those of low socioeconomic status show an accelerated linear growth until the18th month of life, leading to an overcompensation of their initial height deficit. The long-term consequences of these findings remain unclear and require further study.

## Introduction

Height is a widely accepted marker of population health [1]. Many studies have shown adult height to be negatively associated with morbidity and mortality from various diseases [2]–[5]. For example, in a large study among Scottish people, height was found to be inversely associated with all cause mortality, and mortality from respiratory, cardiovascular disease and cancer [2]. A study using data from 4 Scandinavian twin studies to assess the association between height and death from coronary heart disease found that even within twin pairs discordant for height and coronary heart disease, height was inversely associated with risk for death from coronary heart disease [6]. This suggests that the association between height and coronary heart disease risk is due to environmental factors influencing childhood growth. Based on these findings the link between height and health is believed to be founded on circumstances in early life, as growth in childhood is considered a proxy of early life environmental conditions [2]. The first two years of life are particularly critical for height development; they form the period of fastest growth in the entire postnatal life span, meaning that factors negatively influencing growth would have the greatest impact during this window of time [7], [8]. Furthermore, poor growth in the first two years of life has been shown to track into adulthood; undernutrition in early life has been shown to be strongly associated with shorter adult height and also with economic outcomes such as lower human capital [9].

One environmental factor that is associated with height is socioeconomic status; the lower one's educational or income level, the shorter one's attained height [10]. The shorter height is likely to be due to a smaller size at birth, a slower linear growth during childhood, or both. While low socioeconomic status is known to be associated with a smaller birth size [11], much less is known on its association with linear growth during early postnatal life. A positive association between socioeconomic status and height has been demonstrated in children in high-income countries as well as in low- to middle-low income countries [12]–[17], but only a few studies examined the effect of socioeconomic status on height in infants and toddlers, most of which were based on cross-sectional analyses [13], [16], [18], [19]. Investigating the association between socioeconomic status and growth trajectories, however, requires longitudinal analyses of repeated height measurements. Studying this association in the first years of life would indicate whether the development of socioeconomic inequalities in adult height can be partly attributed to inequalities in linear growth during this critical period. Therefore, we studied maternal educational level as a measure of socioeconomic status in relation to repeatedly measured height in children aged 0–2 years, using data from a population-based cohort study performed in a high-income western country. We hypothesized that a low maternal education is associated with a slower linear growth in early childhood. Furthermore, we included other determinants of early postnatal growth to examine to what extent they contribute as mediators to any socioeconomic differences in early growth.

## Methods

### The Generation R Study

This study was embedded within The Generation R Study, a population-based prospective cohort study from fetal life until young adulthood that has previously been described in detail [20], [21]. The Generation R Study is conducted in Rotterdam, the second largest city in the Netherlands. The study area was well defined by postal codes and covered more than half of the city's inhabitants. Ideally, enrolment took place in early pregnancy, but was possible until the birth of the child. All children were born between April 2002 and January 2006 and form a prenatally recruited birth-cohort. Of all eligible children in the study area, 61% participated in the study [21].

### Ethics Statement

The study was conducted in accordance with the guidelines proposed in the World Medical Association Declaration of Helsinki and has been approved by the Medical Ethical Committee of the Erasmus MC, University Medical Center Rotterdam. Written consent was obtained from all participating parents.

### Population for analyses

Out of the 7893 mothers and their children who participated in the postnatal cohort, 6969 had been included prenatally.

In studying socioeconomic disparities in child health, ethnicity is probably the strongest factor that might cause distortion of the apparent effect of socioeconomic status. It has been shown to interact with ethnicity regarding their effects on growth and health [18], [22], [23], and growth patterns may differ by ethnicity [24], [25]. To avoid this type of distortion we restricted our analyses to the subgroup with mothers of Dutch ethnicity [26]. Of the 6969 mothers, 3478 were of Dutch ethnicity ánd gave consent for receiving questionnaires. We excluded twins (n = 90), and the second or third child (n = 327) of the same mother, since data were correlated. We also excluded participants without information on maternal educational level (n = 16) and those without height measurements (n = 73), leaving a study population of 2972 mothers and their children.

### Maternal educational level

Using a questionnaire at enrolment, we established mother's highest achieved education, and categorized this according to the Dutch standard classification into: 1.) high (university or higher), 2.) mid-high (higher vocational training), 3.) mid-low (more than three years of general secondary school, or intermediate vocational training completed), and 4.) low education (no education, primary school, lower vocational training, intermediate general school, or three years or less of general secondary school) [27].

### Height measurements

We collected height measurements that were taken from our participants during regular visits to the Child Health Centers around the ages 1, 2, 3, 4, 6, 11, 14, 18, and 24 months. At the Child Health Centers, growth measurements of those children known to participate in The Generation R Study were written on separate forms, and collected by Generation R employees. Up to and including the second birthday, height was measured to the nearest millimeter using a neonatometer with the child in supine position. After the second birthday, height was measured in standing position. Length at birth was not available, since this was not routinely measured in healthy-born neonates.

### Covariates

**Figure 1:**
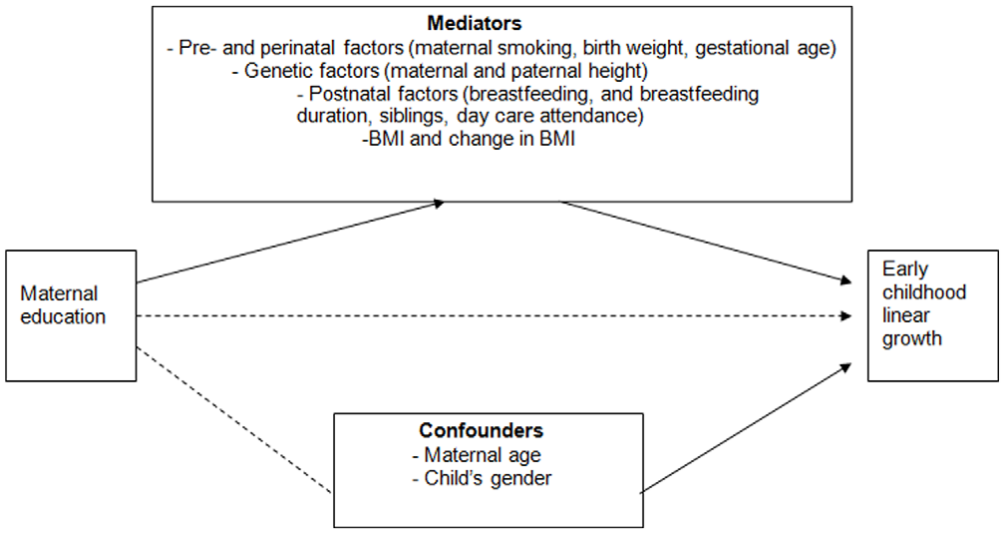
Theoretical model of pathways by which maternal education might influence early childhood linear growth.

In this study we assumed that maternal educational level does not have a direct effect on growth, but rather acts through more proximal determinants of early growth that are unequally distributed across educational subgroups; these determinants are called mediators [28]. Therefore, we evaluated to what extent known determinants of early growth [29]–[32] mediate or ‘explain’ any differences in growth between educational subgroups (figure 1). These determinants are listed below:

Information on whether mother s*moked during pregnancy* (no, yes) was assessed through questionnaires during pregnancy. *Birth weight* and *gestational age at birth* were obtained from midwife and hospital registries. *Maternal and paternal height* was measured at our research centers. Information on *breastfeeding at 2 months* (yes, no) and *breastfeeding duration* (never breastfed, <4 months, 4–6 months, ≥6 months) was derived from questionnaires that were distributed at the child's age of 2, 6, and 12 months. The presence of older *siblings* was established when the child was 6 months old. Information on *day-care attendance* was collected at the ages 6, 12 and 24 months.

Because it has been suggested that body mass or fatness partly regulates linear growth [33], [34], we additionally evaluated the contribution of the child's body mass index (BMI) at time of height measurement, as well as the change in BMI during the preceding periods. BMI was calculated from height and weight (weight/height^2^); weight measurements took place at the same ages as the height measurements.

*Maternal age* at enrolment, and *gender* were treated as potential confounders.

### Statistical analyses

Because the height measurements peaked around the ages 2, 6, 14 and 25 months, they were organized into four measurement points at 2 months (mid-90% range 1.0–3.9 months), 6 months (mid-90% range 5.6–11.4 months), 14 months (mid-90% range 13.7–17.9 months) and 25 months of age (mid-90% range 23.6–29.6 months). For each subject, standard-deviation scores (SDS) were calculated using internally derived gender-specific means and standard deviations: SDS = (measurement – population mean)/population standard deviation).

Using linear regression analyses, we estimated the average height at each age in each educational subgroup adjusted for the child's age at measurement.

Next, we analyzed the association between maternal education and linear growth velocity using linear mixed models [35]. The best fitting model to predict height as a function of age was built using fractional polynomials [36]. To this model we added educational level as a main determinant (reference: high education), and an interaction term of educational level with age. The best-fitting model structure was:
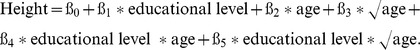



Differences in linear growth velocity between levels of maternal education were then calculated using the derivative of the above model.

Finally, the contribution of covariates to differences in height between educational levels was evaluated by adding these covariates to the linear regression models, first separately, then simultaneously (full model). Then, the full model was additionally adjusted for BMI and the change in BMI between 2 and 6 months, between 6 and 14 months, and between 14 and 25 months. We adjusted for only those covariates that were independent predictors of height when all other covariates were accounted for. For each covariate, an interaction term with educational level was tested for significance. Because missing data on the covariates were not completely random (the proportion of missing values tended to be higher in the lower educational subgroups), complete-case-analysis was likely to introduce biased results. To handle missing values in the covariates (see table 1) we applied multiple imputation based on five imputed data sets (‘PROC MI’ procedure in SAS 9.1.3) [37]. Imputations were based on the relationships between all covariates included in this study.

**Table 1:** General characteristics of the study population (n = 2972)*.

		Maternal educational level				
	Total N = 2972	High N = 1029 (34.6%)	Mid-high N = 793 (26.7%)	Mid-low N = 735 (24.7%)	Low N = 415 (14.0%)	P for trend
**Maternal characteristics**						
Age at enrolment (yrs)	31.5 (4.3)	33.0 (3.2)	32.0 (3.7)	30.4 (4.6)	28.9 (5.5)	<0.001
Nulliparous (%)	65.5	65.1	68.3	67.5	57.8	0.098
Smoking during pregnancy (%)	25.0	14.2	20.8	29.5	51.0	<0.001
Height (cm)	170.9 (6.4)	171.4 (6.1)	171.4 (6.3)	171.8 (6.4)	169.0 (6.9)	<0.001
Height father (cm)	184.1 (7.2)	184.9 (6.9)	184.1 (6.9)	183.6 (7.4)	182.6 (7.5)	<0.001
**Child characteristics**						
Gender (% boys)	50.3	50.6	49.4	48.0	54.9	0.520
Birth weight (g)	3492.6 (545.8)	3552.9 (517.8)	3504.1 (541.2)	3457.2 (564.1)	3383.5 (569.1)	<0.001
Gestational age at birth (weeks)	40.3 (36.0,42.4)	40.3 (36.3,42.4)	40.3 (36.1,42.4)	40.1 (35.7,42.4)	40.0 (34.9,42.3)	<0.001
Breastfeeding at 2 months (%)	66.7	81.4	72.6	54.4	35.2	<0.001
Breastfeeding duration						<0.001
Never	11.6	4.6	6.9	18.2	27.6	
<4 months	45.3	38.3	42.9	52.3	55.8	
4–6 months	12.1	16.3	14.2	8.7	2.6	
≥6 months	31.0	40.8	36.0	20.8	14.0	

		Maternal educational level				
	Total N = 2972	High N = 1029 (34.6%)	Mid-high N = 793 (26.7%)	Mid-low N = 735 (24.7%)	Low N = 415 (14.0%)	P for trend¶
Siblings (% yes)	31.0	32.5	29.4	28.6	35.4	0.852
Day care at 12 months (% yes)	68.7	89.2	71.4	51.4	28.2	<0.001
Day care at 24 months (% yes)	76.9	91.6	78.4	65.0	47.1	<0.001
BMI (kg/m^2^)						
2 months	15.8 (1.5)	15.8 (1.4)	15.8 (1.5)	15.8 (1.5)	15.7 (1.4)	0.227
6 months	17.2 (1.3)	17.1 (1.3)	17.1 (1.3)	17.2 (1.4)	17.2 (1.4)	0.596
14 months	17.1 (1.3)	17.2 (1.3)	17.1 (1.3)	17.1 (1.4)	17.0 (1.3)	0.014
25 months	16.5 (1.3)	16.6 (1.3)	16.5 (1.3)	16.4 (1.4)	16.5 (1.5)	0.019
Height (cm)						
2 months	57.7 (3.6)	57.9 (3.5)	57.6 (3.6)	57.9 (3.7)	57.1 (3.8)	0.003
6 months	68.8 (3.7)	68.8 (3.6)	68.7 (3.6)	68.9 (3.8)	68.9 (4.0)	0.510
14 months	78.5 (2.9)	78.4 (2.8)	78.4 (3.0)	78.7 (3.0)	78.8 (3.0)	0.021
25 months	88.7 (3.6)	88.6 (3.5)	88.5 (3.5)	88.8 (3.6)	88.9 (3.7)	0.095

Yrs: years; cm: centimeters; g: grams; BMI: body mass index; kg: kilograms; m: meters.

* Data were missing for parity (n = 3), smoking during pregnancy (n = 201), breast feeding at 2 mo (n = 237) breast-feeding duration (n = 564), siblings (n = 974), day-care attendance at 12 mo (n = 617), day-care attendance at 24 mo (n = 591), maternal height (n = 3), paternal height (n = 434), BMI 2 months (n = 359), BMI 6 months (n = 132), BMI 14 (n = 295), BMI 25 months (n = 549), height 2 months (n = 359), height 6 months (n = 192), height 14 months (n = 293), height 25 months (n = 545).

¶ P values for trend are derived from x^2^ test for trend (categorical factors) or for the linear trend test of the 1-way analysis of variance.

Because the effect of educational level on growth velocity did not differ by gender (p for interaction education*age*gender >0.4), results were not stratified by gender. Statistical analyses were performed using Statistical Package of Social Sciences version 15.0 for Windows (SPSS Inc, Chicago, IL, USA) and the Statistical Analysis System (SAS) for Windows (SAS Institute Inc, USA), version 9.1.3. A p-value of <0.05 was taken to indicate statistical significance.

## Results

Of the 2972 children, 34.6% of their mothers were low-educated, and 14.0% were high-educated (table 1). Compared with high-educated women, low-educated women were younger, shorter, and were more likely to smoke during pregnancy. Their children were on average lighter at birth, were less likely to be breastfed, and were less likely to go to day care (p for trend all <0.05; table 1).

### Maternal educational level and linear growth

**Figure 2:**
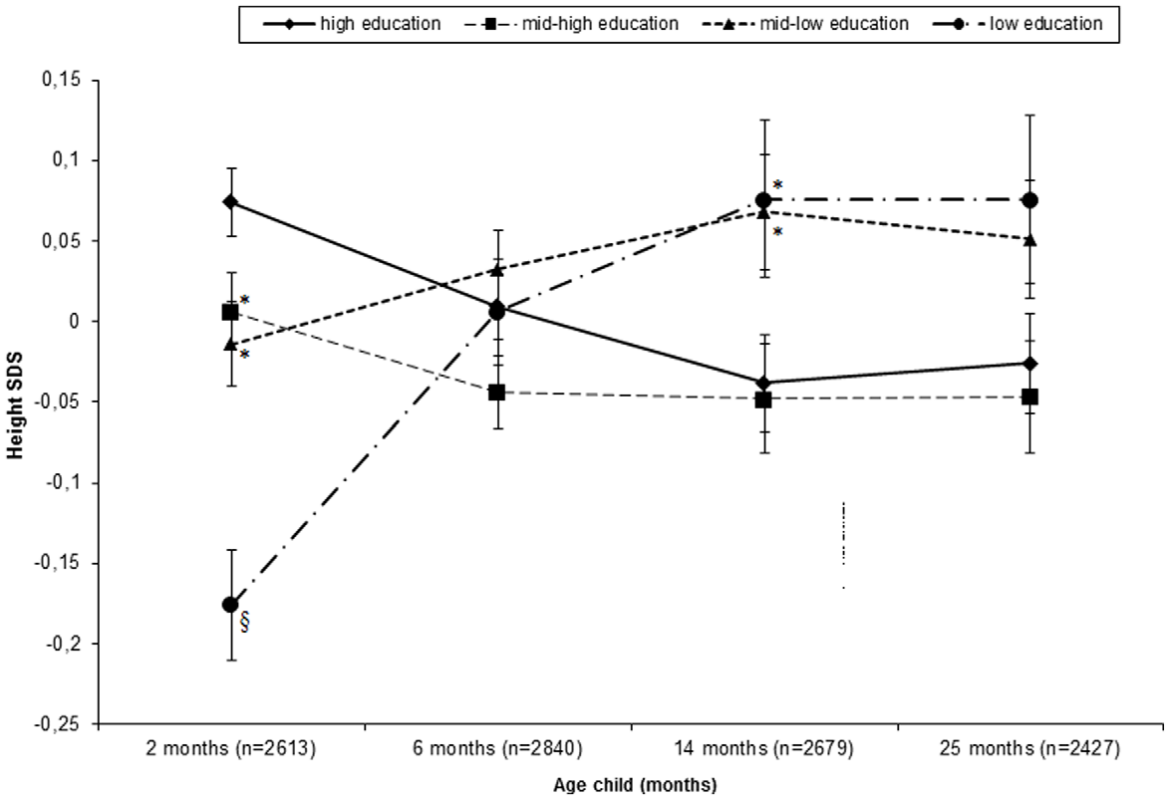
Internally derived SDS +/− standard errors for height, stratified by maternal educational level. All Values adjusted for the child's age at measurement. * Significantly different from height SDS in the high-education subgroup at level p<0.001. § Significantly different from height SDS in the high-education subgroup at level p<0.05.

Compared with children of high-educated mothers, those of low-educated mothers were shorter at 2 months (p<0.001; figure 2). After 2 months, children of mothers with a low educational level showed a relative catch-up growth, while those of mothers with a high level showed a relative catch-down growth. At 6 months there were no differences in height between educational subgroups, but by 14 months, children of mothers with a low educational level were taller than those of mother with a high level (p = 0.046). This difference was no longer statistically significant at 25 months (p = 0.089).

**Figure 3:**
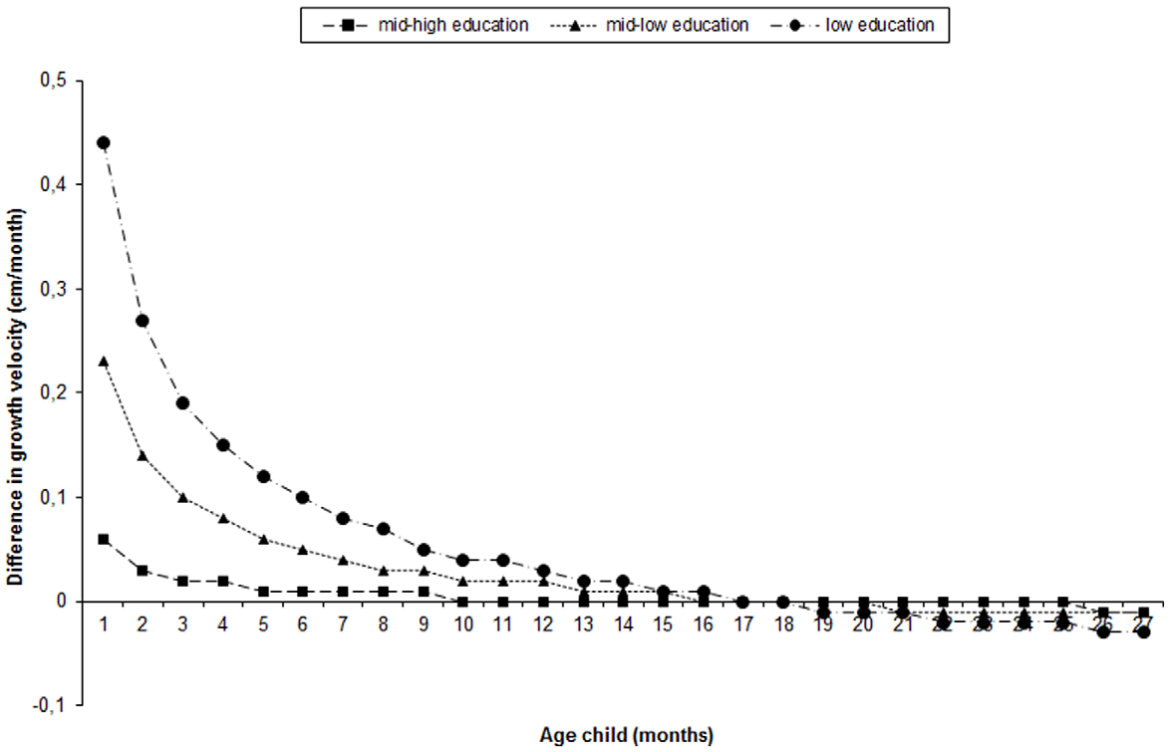
Difference in linear growth velocity, with children of mothers with high education as reference group (n = 2972). Growth curves are derived from linear mixed models. Difference in growth velocity = ß_1_*educational level+ß_2_*0.5*1/√age*educational level.

The linear mixed models indicated differences in growth velocity between educational subgroups (p for educational-level*age and educational-level*√age interactions <0.001). Between 1 and 18 months of age, children of mothers with a low or mid-low educational level grew faster than those of mothers with a high level (figure 3). This difference in growth velocity became smaller with increasing age, and by the 19^th^ month there was no difference in growth velocity. After the 20^th^ month, the association between educational level and linear growth velocity reversed; children of mothers with a low educational level tended to have a slower growth than those of mothers with a high level.

### Contribution of covariates

Table 2 presents the contribution of covariates to the differences in height (in centimeters) between educational subgroups at different ages. Gender, maternal age and siblings were not included in these models, since there were no educational differences in gender or presence of siblings (see table 1) and since maternal age was not an independent predictor of height at any age (data not shown).

**Table 2:** Differences in child's height at 2, 6, 14 and 25 months of age between maternal educational levels*.

	Maternal educational level			
Models	High education	Mid-high education	Mid-low education	Low education
	**2 months (n = 2613)**			
Model 1	Reference	−0.25 (−0.48,−0.01) p: 0.0396	**−0.35 (−0.59,−0.11) p: 0.0045**	**−0.87 (−1.16,−0.58) p<0.0001**
Model 1+ smoking in pregnancy, birth weight & gestational age	Reference	−0.09 (−0.25,0.07) p: 0.2950	0.02 (−0.15,0.19) p: 0.8271	−0.17 (−0.38,0.04) P: 0.1342
Model 1+ maternal and paternal height	Reference	−0.19 (−0.41,0.03); p: 0.0992	−0.20 (−0.43,0.03) p: 0.0793	**−0.43 (−0.71,−0.15) p: 0.0020**
Model 1+ breastfeeding at 2 months	Reference	−0.22 (−0.46,0.01); p: 0.0615	**−0.28 (−0.53,−0.03) p: 0.0251**	**−0.74 (−1.05,−0.43) p<0.0001**
Full model^1^	Reference	−0.06 (−0.22,0.10); p: 0.4714	0.09 (−0.07,0.26) p: 0.2796	0.06 (−0.15,0.28) p: 0.6025
Full model^1^ + BMI at 2 months	Reference	−0.05 (−0.21,0.11) p: 0.5384	0.09 (−0.08,0.26) p: 0.2941	0.04 (−0.17,0.25) p: 0.6966
	**6 months (n = 2840)**			
Model 1	Reference	−0.22 (−0.45,0.01) p: 0.0629	0.03 (−0.21,0.27) p: 0.7977	0.06 (−0.23,0.34) p: 0.7008
Model 1+ smoking in pregnancy, birth weight & gestational age	Reference	−0.10 (−0.31,0.10) p: 0.3139	**0.24 (0.03,0.446) p: 0.0224**	**0.43 (0.16,0.69) p: 0.0015**
Model 1+ maternal and paternal height	Reference	−0.13 (−0.35,0.08) p: 0.2191	0.21 (−0.01,0.43) p: 0.0638	**0.51 (0.24,0.78) p: 0.0002**
Model 1+ breastfeeding duration	Reference	−0.24 (−0.47,0.01) p: 0.0391	−0.08 (−0.33,0.16) p: 0.4961	−0.10 (−0.40,0.20) p: 0.5095
Model 1+ day-care attendance 6 months	Reference	−0.24 (−0.47,−0.001) p: 0.0491	−0.001 (−0.25,0.25) p: 0.9920	0.001 (−0.32,0.32) p: 0.9968
Full model^2^	Reference	−0.14 (−0.34,0.06) p: 0.1657	0.09 (−0.13,0.31) p: 0.4157	**0.34 (0.06,0.63) p: 0.0177**
Full model^2^ + BMI at 6 months	Reference	−0.14 (−0.34,0.06) p: 0.1586	0.09 (−0.13,0.31) p: 0.4194	**0.33 (0.05,0.61) p: 0.0220**
Full model^2^ + change in BMI 2–6 months	Reference	−0.15 (−0.34,0.05) p: 0.1371	0.08 (−0.14,0.29) p: 0.4884	**0.33 (0.05,0.61) p: 0.0212**
	**14 months (n = 2679)**			
Model 1	Reference	−0.04 (−0.30,0.22) p: 0.7719	**0.28 (0.007,0.54) p: 0.0441**	**0.40 (0.08,0.72) p: 0.0153**
Model 1+ smoking in pregnancy, birth weight & gestational age	Reference	0.04 (−0.20,0.28) p: 0.7592	**0.44 (0.19,0.70) p: 0.0005**	**0.77 (0.45,1.08) p<0.0001**
Model 1+ maternal and paternal height	Reference	0.03 (−0.21,0.26) p: 0.8287	**0.46 (0.21,0.71) p: 0.0002**	**0.95 (0.65,1.25) p<0.0001**
Model 1+ breastfeeding duration	Reference	−0.05 (−0.31,0.20) p: 0.6858	0.21 (−0.06,0.49) p: 0.1327	0.31 (−0.02,0.65) p: 0.0694
Model 1+ day-care attendance 12 months	Reference	−0.14 (−0.40,0.13) p: 0.3056	0.07 (−0.22,0.36) p: 0.6198	0.07 (−0.30,0.44) p: 0.7097
Full model 3	Reference	−0.07 (−0.30,0.16) p: 0.5353	0.20 (−0.05,0.46) p: 0.1136	**0.61 (0.26,0.95) p: 0.0006**
Full model 3 + BMI at 14 months	Reference	−0.07 (−0.31,0.16) p: 0.5271	0.20 (−0.05,0.46) p: 0.1175	**0.60 (0.26,0.95) p: 0.0006**
Full model 3 + change in BMI 2–6 months	Reference	−0.07 (−0.30,0.16) p: 0.5394	0.21 (−0.05,0.46) p: 0.1112	**0.61 (0.26,0.95) p: 0.0005**
Full model 3 + change in BMI 6–14 months	Reference	−0.08 (−0.31,0.15) p: 0.4726	0.18 (−0.07,0.44) p: 0.1549	**0.60 (0.26,0.94) p: 0.0007**
	**25 months (n = 2427)**			
Model 1	Reference	−0.08 (−0.41,0.25) p: 0.6206	0.25 (−0.09,0.59) p: 0.1472	0.40 (−0.02,0.83) p: 0.0613
Model 1+ smoking in pregnancy, birth weight & gestational age	Reference	−0.01 (−0.32,0.30) p: 0.9430	**0.42 (0.09,0.75) p: 0.0117**	**0.72 (0.30,1.14) p: 0.0007**
Model 1+ maternal and paternal height	Reference	−0.01 (−0.31,0.28) p: 0.9261	**0.49 (0.19,0.80) p: 0.0017**	**1.11 (0.72,1.50) p<0.0001**
Model 1+ breastfeeding duration	Reference	−0.09 (−0.41,0.24) p: 0.6098	0.24 (−0.11,0.59) p: 0.1810	0.38 (−0.06,0.82) p: 0.0910
Model 1+ day-care attendance 24 months	Reference	−0.12 (−0.45,0.22) p: 0.4940	0.19 (−0.17,0.54) p: 0.3053	0.30 (−0.16,0.75) p: 0.2064
Full model^4^	Reference	−0.04 (−0.33,0.25) p: 0.7977	**0.42 (0.09,0.74) p: 0.0122**	**1.00 (0.57,1.43) p<0.0001**
Full model^4^+BMI at 25 months	Reference	−0.05 (−0.34,0.24) p: 0.7443	**0.40 (0.07,0.72) p: 0.0175**	**0.99 (0.57,1.42) p<0.0001**
Full model^4^+change in BMI 2–6 months	Reference	−0.04 (−0.33,0.26) p: 0.8015	**0.42 (0.09,0.74) p: 0.0123**	**1.00 (0.57,1.42) p<0.0001**
Full model^4^+change in BMI 6–14 months	Reference	−0.01 (−0.30,0.28) p: 0.9448	**0.46 (0.14,0.79) p: 0.0049**	**1.03 (0.61,1.46) p<0.0001**
Full model^4^+change in BMI 14–25 months	Reference	−0.06 (−0.34,0.23) p: 0.6970	**0.40 (0.06,0.70) p: 0.0201**	**1.01 (0.59,1.43) p<0.0001**

* Values are differences in centimeters (with 95% CI and p-values) and derived from linear regression analyses performed on the data after applying multiple imputation.

Model 1: adjusted only for child age at measurement.

1 Adjusted for child age at measurement, smoking in pregnancy, birth weight & gestational age, maternal and paternal height, and breastfeeding at 2 months.

2 Adjusted for child age at measurement, smoking in pregnancy, birth weight & gestational age, maternal and paternal height, breastfeeding duration, and day-care attendance at 6 months.

3 Adjusted for child age at measurement, smoking in pregnancy, birth weight & gestational age, maternal and paternal height, breastfeeding duration, and day-care attendance at 12 months.

4 Adjusted for child age at measurement, smoking in pregnancy, birth weight & gestational age, maternal and paternal height, breastfeeding duration, and day-care attendance at 24 months.

At 2 months, the variables smoking during pregnancy, birth weight and gestational duration contributed most to the shorter height of children in the lowest educational subgroup compared with the highest; adjustment for these factors together reduced the difference in height from −0.87 cm (95% CI: −1.16,−0.58) to −0.17 cm (95% CI: −0.38,0.04). In the full model, the differences in height disappeared.

By 14 months, children of mothers with a low educational level were 0.40 cm taller (95% CI: 0.08,0.72) than those of mothers with a high level. This difference became even stronger after adjustment for smoking during pregnancy, birth weight and gestational duration, and for maternal and paternal height. In contrast, adjustment for day-care attendance and breastfeeding explained part of the taller height. In the full model, children in the lowest educational subgroup were still significantly taller than those in the highest subgroup (difference: 0.60 cm; 95% CI: 0.26,0.94). We found comparable results at 25 months of age; children in the lowest educational subgroup were then 1.01 cm taller (95% CI: 0.59,1.43) in the full model.

Adding ‘BMI’ or ‘change in BMI’ to the full models did not influence the effect estimates.

## Discussion

Our study showed that compared with children of mothers with a high education, those of mothers with a low education were shorter at the age of 2 months. However, their height deficit was overcompensated by a faster linear growth between 1 and 18 months of age. By 14 months, children in the lowest educational subgroup were even taller than those in the highest educational subgroup.

### Socioeconomic status and early linear growth

Several previous studies have investigated the association between socioeconomic status and height development in infants and toddlers [13], [16], [18], [19]. For example, Seguin et al [19] found that longstanding material hardship increased the risk of having a height under the tenth percentile at the age of 2.5 years, suggesting that the socioeconomic gradient in height may arise during the first years of life. A recent study based on data from the ALSPAC study, a British cohort study, showed a positive relationship between level of maternal education and child's height from birth to 10 years of age [16]. Height inequalities in childhood were also clearly present in the USA [15]. In our study, height at the age of 2 months was associated with maternal educational level in the expected direction: the lower the educational level, the shorter the offspring's height.

An unexpected finding was the faster linear growth during the first 1.5 years associated with a low maternal education. However, this phenomenon has been reported before: among infants in whom height was measured between 0 and 2 years, Herngreen et al [18] found that children of low socioeconomic status tended to be initially shorter, but had a higher gain in height after birth compared with children of high socioeconomic status. In contrast to our study, however, socioeconomic status was no longer associated with height or change in height after allowing for other factors, i.e. ethnic descent of the parents, gestational age, birth weight, parity, maternal smoking during pregnancy, maternal age and height of the parents. In another study from the UK by Howe et al, the data suggest that between the age of 2 and 11 months, daughters of mothers with low education grow faster than daughters of mothers with a high education. However, after this age the relationship between maternal educational level and growth velocity reversed [16].

We considered different mechanisms driving the associations between a lower maternal educational level and a faster linear growth and taller height by 14 months of age.

The first is selection bias. Although the participation in The Generation R Study was relatively high (61%; 68% for participants with a Dutch ethnicity) [21], [38], there was some selection towards a study population that was relatively highly educated and more healthy [21]. For example, compared to our study population mean birth weight is somewhat lower in the general Dutch population (3434 vs. 3492 grams), and the number of children that are never breastfed is higher (25% vs. 12%). For selective participation to explain our results, non-participants would have to have been more often of low socioeconomic status with children who are relatively short and grow relatively slow. This is difficult to ascertain, but is unlikely to fully explain our results. Additionally, 18% of the participants who were eligible for inclusion in our study were lost to follow-up. Compared to participants included in present analyses, children lost to follow-up were lighter at birth, and had mothers who were less educated and more likely to smoke during pregnancy (data not shown). The effect of this selection on our effect estimates depends on the presence or absence of postnatal catch-up growth in children lost to follow-up. The mechanisms that signal and regulate postnatal catch-up growth are not completely understood, but previous studies suggest that intra-uterine growth restricted children, such as seen in children of mothers who smoked during pregnancy, tend to show a compensatory postnatal catch-up growth [32]. If this is also the case for those children lost to follow-up, this would have led to an underestimation of our effect estimates

Second, the relatively faster growth might be a biological response to adverse intrauterine exposures. Children of low socioeconomic status were more likely to have mothers who smoked during pregnancy and were smaller at birth. Accelerated postnatal growth is often seen in children born to smoking mothers or born relatively small [32], [39]. However, in our study, adjustment for maternal smoking rates, birth weight and gestational age did not explain the taller height in lower educational subgroups. Instead, it exacerbated the difference in height. In other words, even when adverse intra-uterine circumstances, which are associated with a tendency for postnatal catch-up growth, are taken into account, children of low-educated mothers would still be taller than children of high-educated mothers. This suggest that postnatal factors play a more important role in explaining the taller height in children of mothers with a low educational level versus those of mothers with a high educational level.

Indeed, our results suggest that socioeconomic differences in feeding practices, another major determinant of early growth [29], might explain the differences in linear growth. At 14 months, part of the taller stature in the subgroup of low education was explained by a shorter breastfeeding duration in this subgroup. It is known that breastfeeding is less common in lower socioeconomic subgroups [40]. It is also known that compared to bottle-fed infants, breastfed infants grow slower in the first year of life, causing bottle-fed infants to be heavier and taller than their breastfed counterparts after the age of 6 months [29], [41]. This may be due to excessive feeding or a higher nitrogen and energy intake of formula-fed infants [42], [43].

The low rate of day-care attendance in children of mothers with a low education also contributed to their taller height, because in our data day-care attendance was associated with a slower linear growth (data not shown). We found no previous studies that investigated the specific effect of day-care attendance on early growth to support this finding. Frequent infections or a lower risk of overfeeding might underlie this association [31], [43].

After taking all covariates into account, children in the lowest educational subgroup were about 1 cm taller than those in the highest educational subgroup. This is likely to be explained by other growth-stimulating factors that were not available for this study. We believe that nutritional factors and total amount of energy intake are the most important factors that might explain the remaining differences in height between children of low and high-educated mothers. This merets further investigation.

One might wonder: what is the clinical significance of a 1 cm difference in height? On an individual level, a difference of 1 cm seems of little importance. However, since the difference in height is a result of a difference in linear growth velocity, a more important question to consider is: Is the observed acceleration in linear growth in children of low-educated mothers beneficial to them? It seems to be, at least on the short term. Due to this acceleration in growth, infants of low–educated mothers were able to compensate their initial height deficit. However, there is reason to believe that, in the long run, the accelerated growth might have adverse health consequences. Population-based studies as well as studies in subjects born preterm or small for gestational age, have shown that accelerated growth during childhood, both in weight and in height, is associated with later cardiovascular disease and its risk factors, including insulin insensitivity, obesity and higher blood pressure [44]–[51]. These effects were independent of size at birth, suggesting that accelerated growth, rather than intrauterine growth retardation adversely programs later cardiovascular outcomes, shifting the focus away from the so-called “fetal origins hypothesis” of cardiovascular disease to an “accelerated postnatal growth hypothesis” [50], [51]. Given these latest insights, one may speculate that the relative growth retardation in utero, followed by the relative growth acceleration in early childhood observed in children of lower educational subgroups might lead to an increased propensity to later obesity, metabolic syndrome and cardiovascular disease. Such a hypothesis would fit the well-known socioeconomic gradient in cardiovascular disease and its risk factors [52]–[55].

In light of the current obesity epidemic, we have previously reported similar analyses for BMI and overweight as an outcome [56]. In this study, children from mothers with a low socioeconomic status had a lower BMI at 24 months than children from mothers with a high socioeconomic status. There were no differences in BMI before that age. While height was relatively larger at 24 months in children from mothers with a low socioeconomic status, weight was relatively lower, which explained the lower BMI at that age. These results suggest that children from low socioeconomic status are catching up, first in length/height, and the expectation is that weight will follow.

### Methodological considerations and limitations

Socioeconomic status refers to the “social and economic factors that influence what positions individuals or groups hold within the structure of society” [57]. It is a complex and multifactorial construct. Although there are other measures of socioeconomic status, including income level and occupational class [57], [58], we selected maternal educational level as a main indicator for several reasons:

First, educational level partly reflects maternal resources because it structures occupation and income. Because level of education is of great influence on future income, analyses using income level as main socioeconomic indicator may in turn be confounded by educational level, and therefore give biased results [59], [60].

Second, educational level also reflects non-economic social characteristics, such as literacy, problem-solving skills, prestige, general knowledge, and knowledge with respect to health behavior, feeding practices and health of their children [58], [61].

Third, unlike for example occupational class, a classification according to educational attainment can be applied to teenage and unemployed mothers.

Last, educational level is relatively stable over time.

We restricted our analyses to the subgroup with mothers of Dutch ethnicity. About 18% of the children had a father with a non-Dutch ethnicity, causing some heterogeneity in the study population. However, we repeated the analyses in the subgroup of children of whom both parents had a Dutch ethnicity and found the same significant differences.

In our study we were unable to evaluate the impact of change in maternal educational level over time on our results. In theory, it is possible that part of the mothers under study gained a higher educational level at the end of the follow-up period than they had at time of inclusion in the study. In The Generation R Study, maternal educational level was re-assessed at the child's age of 3 years. From these data we can derive that about 8% of the mothers who had a low educational level at time of inclusion, had a higher educational level at the child's age of 3 years (data not shown). This percentage is probably lower at the age of 2 years. If, in contrast with our results, a high maternal educational level is actually associated with a taller height of the child compared to a low maternal educational level, upward change of maternal education with time may have led to an overestimation of our results at the age of 14 months. However, we believe that the proportion of women with a higher educational level at age of 3 years than at inclusion is too small to fully explain our results. It would be interesting to analyse migration in socioeconomic status in relation to child health and development in future studies.

In our study, information on length at birth was not available, since this is not a routine measurement in the Netherlands. Thus, we could not report differences in length at birth between children of low and high-educated mothers. However, the availability of multiple height measurements from 2 months to 24 months, and the use of fractional polynomials and multilevel analysis enabled us to fit a model for predicted height. The absence of an extra measurement point at birth is expected to have little effect on the fitted growth model, and thus little effect on our results.

Caution should be taken when generalizing our findings. The phenomenon of accelerated linear growth during early childhood in children of low socioeconomic status, and in particular the taller height, may be specific to affluent Western populations with increasing availability of inexpensive, energy-dense food. Our findings are probably not generalizable to low or middle-low income countries, where low socioeconomic status is generally associated with a lack of resources for adequate nutrition.

### Conclusions

Our work suggests that, while at the onset of their growth trajectory children of low socioeconomic status are shorter than their peers of high socioeconomic status, they show a relative accelerated linear growth until the18th month of life. This phenomenon might have consequences for their long-term cardiovascular health [47], [48] and should be studied further. Further follow-up is necessary to study how socioeconomic status affects growth after the second year of life, and how this relates to socioeconomic inequalities in adult height and health.

## References

[1] Tanner JM (1992). Growth as a measure of the nutritional and hygienic status of a population.. Horm Res.

[2] Davey Smith G, Hart C, Upton M, Hole D, Gillis C (2000). Height and risk of death among men and women: aetiological implications of associations with cardiorespiratory disease and cancer mortality.. J Epidemiol Community Health.

[3] Gunnell D, Okasha M, Smith GD, Oliver SE, Sandhu J (2001). Height, leg length, and cancer risk: a systematic review.. Epidemiol Rev.

[4] Jousilahti P, Tuomilehto J, Vartiainen E, Eriksson J, Puska P (2000). Relation of adult height to cause-specific and total mortality: a prospective follow-up study of 31,199 middle-aged men and women in Finland.. Am J Epidemiol.

[5] Langenberg C, Shipley MJ, Batty GD, Marmot MG (2005). Adult socioeconomic position and the association between height and coronary heart disease mortality: findings from 33 years of follow-up in the Whitehall Study.. Am J Public Health.

[6] Silventoinen K, Zdravkovic S, Skytthe A, McCarron P, Herskind AM (2006). Association between height and coronary heart disease mortality: a prospective study of 35,000 twin pairs.. Am J Epidemiol.

[7] Fredriks AM, van Buuren S, Hirasing RA, verloove-Vanhorick SP, Wit JM (2001). Voortgaande toename van de lengtegroei bij Nederlandse kinderen in de periode 1955–1997 (Dutch).. Ned Tijdschrift Geneesk.

[8] Tanner JM, Davies PS (1985). Clinical longitudinal standards for height and height velocity for North American children.. J Pediatr.

[9] Victora CG, Adair L, Fall C, Hallal PC, Martorell R (2008). Maternal and child undernutrition: consequences for adult health and human capital.. Lancet.

[10] Cavelaars AE, Kunst AE, Geurts JJ, Crialesi R, Grotvedt L (2000). Persistent variations in average height between countries and between socio-economic groups: an overview of 10 European countries.. Ann Hum Biol.

[11] Mortensen LH, Diderichsen F, Arntzen A, Gissler M, Cnattingius S (2008). Social inequality in fetal growth: a comparative study of Denmark, Finland, Norway and Sweden in the period 1981–2000.. J Epidemiol Community Health.

[12] de Menezes RC, de Lira PI, Leal VS, Oliveira JS, Santana SC (2012). Determinants of stunting in children under five in Pernambuco, northeastern Brazil.. Rev Saude Publica.

[13] Drachler Mde L, Bobak M, Rodrigues L, Aertz DR, Leite JC (2002). The role of socioeconomic circumstances in differences in height of pre-school children within and between the Czech Republic and southern Brazil.. Cent Eur J Public Health.

[14] du Prel X, Kramer U, Behrendt H, Ring J, Oppermann H (2006). Preschool children's health and its association with parental education and individual living conditions in East and West Germany.. BMC Public Health.

[15] Finch BK, Beck AN (2011). Socio-economic status and z-score standardized height-for-age of U.S.-born children (ages 2–6).. Econ Hum Biol.

[16] Howe LD, Tilling K, Galobardes B, Smith GD, Gunnell D (2012). Socioeconomic differences in childhood growth trajectories: at what age do height inequalities emerge?. J Epidemiol Community Health.

[17] Whincup PH, Cook DG, Shaper AG (1988). Social class and height.. Bmj.

[18] Herngreen WP, van Buuren S, van Wieringen JC, Reerink JD, Verloove-Vanhorick SP (1994). Growth in length and weight from birth to 2 years of a representative sample of Netherlands children (born in 1988–89) related to socioeconomic status and other background characteristics.. Ann Hum Biol.

[19] Seguin L, Xu Q, Gauvin L, Zunzunegui MV, Potvin L (2005). Understanding the dimensions of socioeconomic status that influence toddlers' health: unique impact of lack of money for basic needs in Quebec's birth cohort.. J Epidemiol Community Health.

[20] Jaddoe VW, Bakker R, van Duijn CM, van der Heijden AJ, Lindemans J (2007). The Generation R Study Biobank: a resource for epidemiological studies in children and their parents.. Eur J Epidemiol.

[21] Jaddoe VW, van Duijn CM, van der Heijden AJ, Mackenbach JP, Moll HA (2008). The Generation R Study: design and cohort update until the age of 4 years.. Eur J Epidemiol.

[22] Braveman P, Cubbin C, Marchi K, Egerter S, Chavez G (2001). Measuring socioeconomic status/position in studies of racial/ethnic disparities: maternal and infant health.. Public Health Rep.

[23] Dowd JB, Zajacova A, Aiello A (2009). Early origins of health disparities: burden of infection, health, and socioeconomic status in U.S. children.. Soc Sci Med.

[24] Fredriks AM, van Buuren S, Jeurissen SE, Dekker FW, Verloove-Vanhorick SP (2003). Height, weight, body mass index and pubertal development reference values for children of Turkish origin in the Netherlands.. Eur J Pediatr.

[25] Fredriks AM, van Buuren S, Jeurissen SE, Dekker FW, Verloove-Vanhorick SP, et al. (204) Height, weight, body mass index and pubertal development references for children of Moroccan origin in The Netherlands.. Acta Paediatr.

[26] Statistics NetherlandsAllochtoneninNederland (2004). Voorburg/Heerlen; 2004.

[27] Statistics NetherlandsStandaardOnderwijsindeling (2003). Voorburg/Heerlen; 2004.

[28] McNamee R (2003). Confounding and confounders.. Occup Environ Med 60(3):227–34; quiz 164,.

[29] Kramer MS, Guo T, Platt RW, Vanilovich I, Sevkovskaya Z (2004). Feeding effects on growth during infancy.. J Pediatr.

[30] Lawson DW, Mace R (2008). Sibling configuration and childhood growth in contemporary British families.. Int J Epidemiol.

[31] Li L, Manor O, Power C (2004). Early environment and child-to-adult growth trajectories in the 1958 British birth cohort.. Am J Clin Nutr.

[32] Ong KK, Preece MA, Emmett PM, Ahmed ML, Dunger DB (2002). Size at birth and early childhood growth in relation to maternal smoking, parity and infant breast-feeding: longitudinal birth cohort study and analysis.. Pediatr Res.

[33] Dewey KG, Hawck MG, Brown KH, Lartey A, Cohen RJ (2005). Infant weight-for-length is positively associated with subsequent linear growth across four different populations.. Matern Child Nutr.

[34] Waterlow JC (1994). Relationship of gain in height to gain in weight.. Eur J Clin Nutr 48 Suppl 1:S72–3; discussion.

[35] Goldstein H (1995). Multilevel statistical models..

[36] Royston P, Ambler G, Sauerbrei W (1999). The use of fractional polynomials to model continuous risk variables in epidemiology.. Int J Epidemiol.

[37] Rubin DB (1987). Multiple Imputation for Nonresponse in Surveys..

[38] Center for Research and Statistics, Rotterdam (COS); http://www.cos.rotterdam.nl; (2005). http://www.cos.rotterdam.nl.

[39] Hokken-Koelega AC, De Ridder MA, Lemmen RJ, Den Hartog H, De Muinck Keizer-Schrama SM (1995). Children born small for gestational age: do they catch up?. Pediatr Res.

[40] Dubois L, Girard M (2003). Social inequalities in infant feeding during the first year of life. The Longitudinal Study of Child Development in Quebec (LSCDQ 1998–2002).. Public Health Nutr.

[41] Spyrides MH, Struchiner CJ, Barbosa MT, Kac G (2008). Effect of predominant breastfeeding duration on infant growth: a prospective study using nonlinear mixed effect models.. J Pediatr (Rio J).

[42] Dewey KG (2003). Is breastfeeding protective against child obesity?. J Hum Lact.

[43] Heinig MJ, Nommsen LA, Peerson JM, Lonnerdal B, Dewey KG (1993). Energy and protein intakes of breast-fed and formula-fed infants during the first year of life and their association with growth velocity: the DARLING Study.. Am J Clin Nutr.

[44] Botton J, Heude B, Maccario J, Ducimetiere P, Charles MA (2008). Postnatal weight and height growth velocities at different ages between birth and 5 y and body composition in adolescent boys and girls.. Am J Clin Nutr.

[45] Fewtrell MS, Doherty C, Cole TJ, Stafford M, Hales CN (2000). Effects of size at birth, gestational age and early growth in preterm infants on glucose and insulin concentrations at 9–12 years.. Diabetologia.

[46] Forsen T, Eriksson JG, Tuomilehto J, Osmond C, Barker DJ (1999). Growth in utero and during childhood among women who develop coronary heart disease: longitudinal study.. Bmj.

[47] Law CM, Shiell AW, Newsome CA, Syddall HE, Shinebourne EA (2002). Fetal, infant, and childhood growth and adult blood pressure: a longitudinal study from birth to 22 years of age.. Circulation.

[48] Leunissen RW, Oosterbeek P, Hol LK, Hellingman AA, Stijnen T (2008). Fat mass accumulation during childhood determines insulin sensitivity in early adulthood.. J Clin Endocrinol Metab.

[49] Parsons TJ, Power C, Manor O (2001). Fetal and early life growth and body mass index from birth to early adulthood in 1958 British cohort: longitudinal study.. Bmj.

[50] Singhal A, Cole TJ, Fewtrell M, Deanfield J, Lucas A (2004). Is slower early growth beneficial for long-term cardiovascular health?. Circulation.

[51] Singhal A, Fewtrell M, Cole TJ, Lucas A (2003). Low nutrient intake and early growth for later insulin resistance in adolescents born preterm.. Lancet.

[52] Colhoun HM, Hemingway H, Poulter NR (1998). Socio-economic status and blood pressure: an overview analysis.. J Hum Hypertens.

[53] Laaksonen M, Talala K, Martelin T, Rahkonen O, Roos E (2008). Health behaviors as explanations for educational level differences in cardiovascular and all-cause mortality: a follow-up of 60 000 men and women over 23 years.. Eur J Public Health.

[54] Langenberg C, Hardy R, Kuh D, Brunner E, Wadsworth M (2003). Central and total obesity in middle aged men and women in relation to lifetime socioeconomic status: evidence from a national birth cohort.. J Epidemiol Community Health.

[55] Manor O, Eisenbach Z, Friedlander Y, Kark JD (2004). Educational differentials in mortality from cardiovascular disease among men and women: the Israel Longitudinal Mortality Study.. Ann Epidemiol.

[56] van Rossem L, Silva LM, Hokken-Koelega A, Arends LR, Moll HA (2010). Socioeconomic status is not inversely associated with overweight in preschool children.. J Pediatr 157(6):929–935.

[57] Lynch J, Kaplan GA (2000). Socioeconomic position.. Social epidemiology.

[58] Galobardes B, Shaw M, Lawlor DA, Lynch JW, Davey Smith G (2006). Indicators of socioeconomic position (part 1).. J Epidemiol Community Health.

[59] Spencer N (2005). Maternal education, lone parenthood, material hardship, maternal smoking, and longstanding respiratory problems in childhood: testing a hierarchical conceptual framework.. J Epidemiol Community Health.

[60] Thrane N, Sondergaard C, Schonheyder HC, Sorensen HT (2005). Socioeconomic factors and risk of hospitalization with infectious diseases in 0- to 2-year-old Danish children.. Eur J Epidemiol.

[61] Braveman PA, Cubbin C, Egerter S, Chideya S, Marchi KS (2005). Socioeconomic status in health research: one size does not fit all.. Jama.

